# Efficacy of a novel self-assembling peptide gel for hemostasis in refractory neoplastic bleeding

**DOI:** 10.1016/j.vgie.2023.04.001

**Published:** 2023-06-12

**Authors:** Yuki Kano, Hironori Sunakawa, Keiichiro Nakajo, Tomohiro Kadota, Tomonori Yano

**Affiliations:** Department of Gastroenterology and Endoscopy, National Cancer Center Hospital East, Kashiwa, Chiba, Japan

## Abstract

Video 1 Outline of how self-assembling peptide gel can be used ex vivo.

Outline of how self-assembling peptide gel can be used ex vivo.

## Case

Anemia is seen on laboratory tests in up to 95% of patients with metastatic cancer, and direct bleeding from advanced tumors in the digestive tract can be seen with endoscopy in predominant cases of GI cancer.^1^ Hemostasis with mechanical hemostatic techniques via endoscopy is often difficult to achieve for advanced GI cancer; therefore, bleeding from advanced tumors is often treated only with blood transfusions, which can often be life-threatening in our practice.

The self-assembling peptide gel (PuraStat; 3-D Matrix, Tokyo, Japan) is an endoscopically delivered hemostatic agent for the treatment of GI bleeding. Self-assembling peptide gel has been reported for use in intraprocedural bleeding and delayed bleeding in endoscopic submucosal dissection (ESD).^2^, ^3^, ^4^, ^5^ There have been no reports on the efficacy of self-assembling peptide gel for palliative hemostasis of a tumor hemorrhage from advanced cancer. We report a case in which the use of self-assembling peptide gel was effective for the management of hemostasis for unresectable metastatic GI cancer.

**Figure 1:**
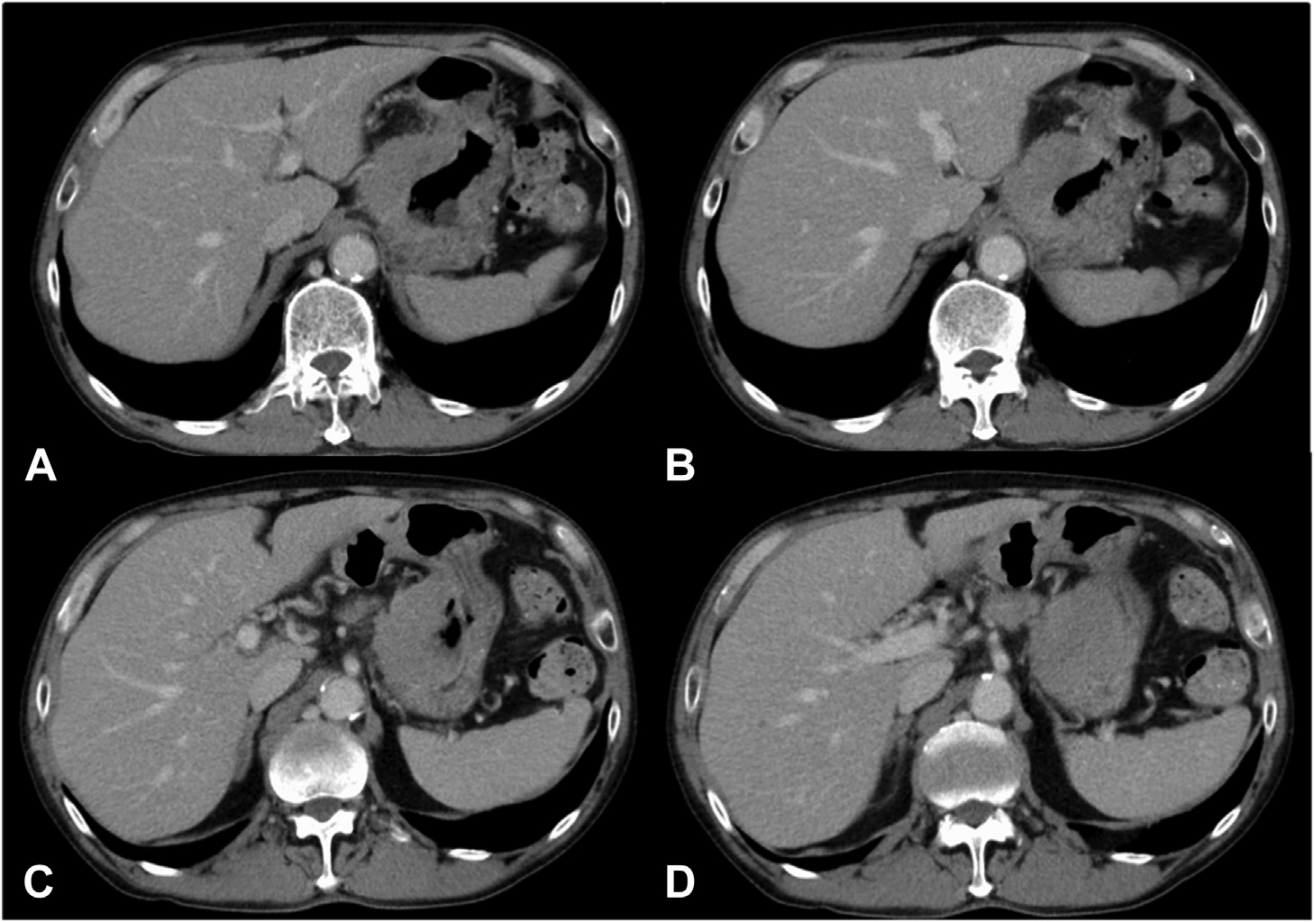
A plain CT scan of the abdomen showed an enlarged lymph node **(C)** on the lesser curvature side invading the stomach.

A 60-year-old male patient underwent ESD for superficial esophageal cancer at our hospital and was pathologically diagnosed with noncurative resection (pT1b-SM2; 600μm, ly1, v1). The patient refused additional treatment and was followed. One year and 2 months after ESD, metastatic recurrence occurred in multiple lymph nodes, and systemic chemotherapy was performed. After 8 courses of FOLFOX, he complained of massive black stools, and laboratory tests showed severe anemia (Hb: 5.0 g/dL). A simple CT scan of the abdomen showed an enlarged lymph node on the lesser curvature side invading into the stomach (Fig. 1). After suspicion of GI bleeding, an emergency endoscopy was performed.

## Procedure

**Figure 2:**
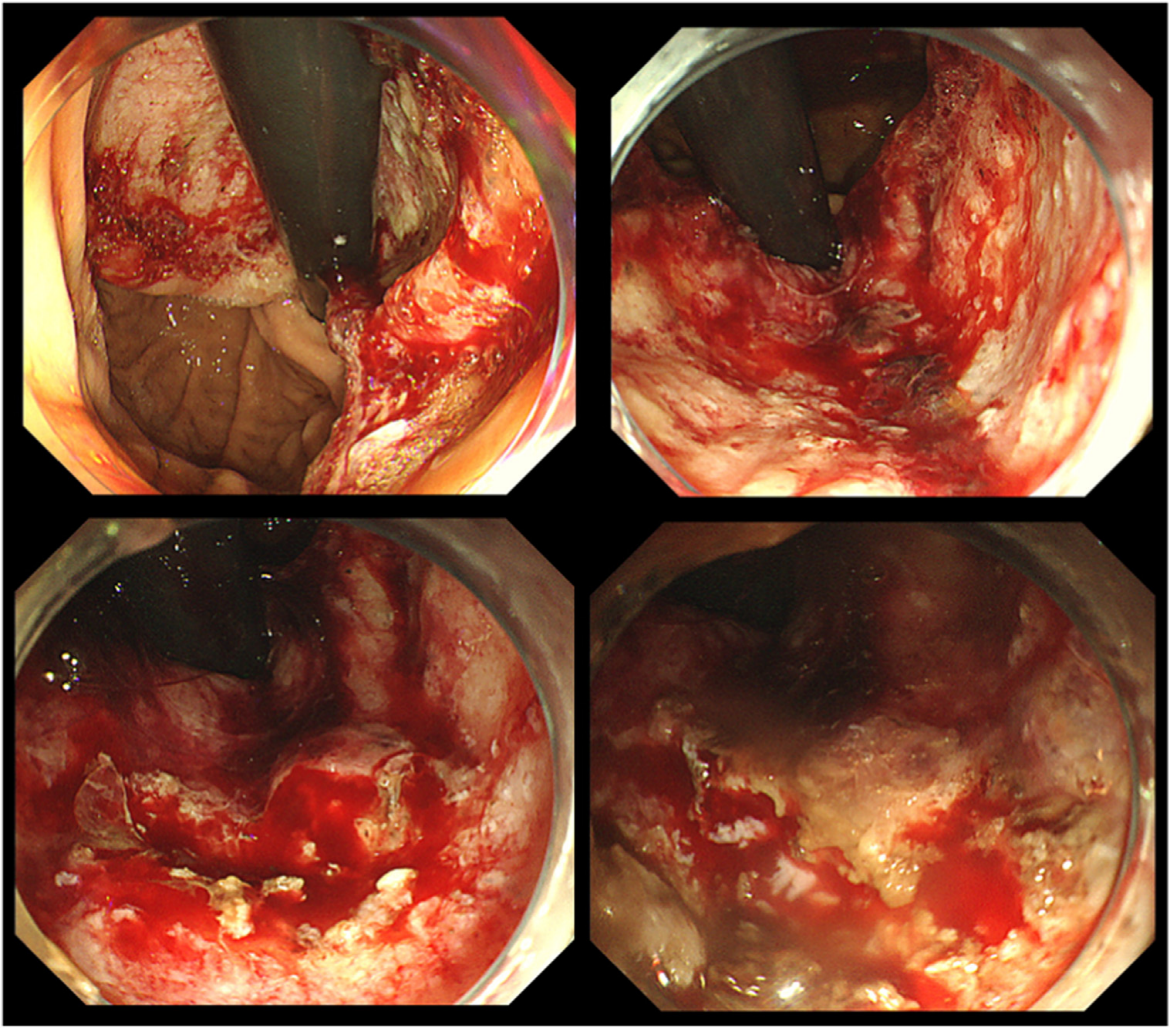
Upper GI endoscopy revealed an ulcerative lesion in the lesser curvature and multiple hemorrhages at the ulcer, which were cauterized with hemostatic forceps. Hemostasis was not achieved.

Upper GI endoscopy revealed an ulcerative lesion in the lesser curvature, which was consistent with its location on a plain CT scan and was diagnosed as gastric invasion of the lymph nodes and multiple hemorrhages at the ulcer, but hemostasis by hemostatic forceps was not achieved (Fig. 2). Self-assembling peptide gel was applied to the bleeding site. Self-assembling peptide gel stabilized the scope by bringing the bottom of the ulcer to 6 o'clock and was applied efficiently and slowly using gravity to prevent clumping, which may cause the gel to become easily dislodged. We took care to prevent blood from adhering to the catheter tip to prevent the protein matrix gel from clogging in the catheter. Hemostasis was confirmed (Video 1, available online at www.videogie.org).

After admission, 4 units of blood transfusion were administered on the first and second days, and their Hb rose to 9.0 g/dL. A proton-pump inhibitor was administered. Since hemostasis with self-assembling peptide gel alone could not be permanent because of the disease progression, palliative irradiation was started for bleeding control on the fifth day. Three months after hemostasis, the disease had progressed but anemia had not progressed, There was no rebleeding or adverse events related to the self-assembling peptide gel.

## Discussion

Self-assembling peptide gel is a novel synthetic self-assembling peptide that is licensed for use as a hemostat. The gel forms an extracellular scaffold matrix when activated by the change in pH that occurs upon contact with blood and forms a stable mechanical barrier over the bleeding site, thereby facilitating intrinsic in vivo hemostasis.^3^

There have been reports of self-assembling peptide gel in the endoscopic field for post-ESD bleeding,^3^^,^^4^^,^^6^ radiation proctitis,^7^ and GI bleeding.^5^^,^^7^, ^8^, ^9^ However, there are no reports of its use for palliative hemostasis of neoplastic bleeding.

Initial preclinical studies have shown additional benefits, including improved wound healing,^3^ and rebleeding after hemostasis for benign peptic ulcers has been reported to be less frequent than with other hemostatic methods.^4^^,^^6^^,^^9^ However, in the case of neoplastic hemorrhage, the wound-healing effect of the self-assembling peptide gel alone may be insufficient, and the risk of rebleeding because of cancer progression is presumed to be high without curative treatment. Therefore, it should be used in combination with palliative irradiation or other oncological treatments. Endoscopic intervention using the self-assembling peptide gel can be a useful bridge therapy prior to palliative irradiation or systemic chemotherapy in the oncological management of advanced GI cancer.

The use of self-assembling peptide gel is limited, however, because it is difficult to apply in the case of arterial eruptive bleeding, as the self-assembling peptide gel is washed away by the blood. In the case of arterial eruptive hemorrhage, it is necessary to consider other modalities for hemostasis.

## Disclosure

*Dr Yano receives honoraria for lecture* *fees* *from 3D Matrix, FUJIFILM, and Olympus. Dr Yano also receives research* *grants* *from Olympus, FUJIFILM, and HOYA PENTAX for the work described in this article. The other authors did not disclose any financial relationships.*

## Supplementary data

Video 1 Outline of how self-assembling peptide gel can be used ex vivo.
